# Travel-associated emissions of intravitreal injections

**DOI:** 10.1007/s00417-025-06963-x

**Published:** 2025-11-01

**Authors:** Denise Yang-Seeger, Annkathrin Schellstede, Laurenz J. B. Pauleikhoff, Martin S. Spitzer, Johannes Birtel

**Affiliations:** 1 Department of Ophthalmology, University Medical Center Hamburg-Eppendorf, Hamburg, Germany; 2 Department of Ophthalmology, University of Bonn, Bonn, Germany

**Keywords:** Sustainability, Intravitreal injections, Travel, Emission, Carbon footprint, Ophthalmology

## Abstract

**Purpose:**

Intravitreal injections (IVIs) are among the most frequently performed ophthalmic procedures and have been associated with a substantial carbon footprint. Travel-associated emissions are considered a key driver of IVIs’ carbon footprint; however, no study in a country with a decentralised healthcare system has assessed IVI-related emissions. Here, the IVI-associated carbon footprint in an urban German tertiary referral centre was investigated.

**Methods:**

In total, 340 patients were included. The carbon footprint was assessed based on the distance travelled by patients and accompanying persons, the mode of transport, and postoperative follow-up visits. Demographic data, treatment indication, and the injected medication were collected.

**Results:**

The average travel-associated CO_2_ equivalent (CO_2_eq) for a single IVI was 11.1 kg. Of this, 9.1 kg CO_2_eq and 2 kg CO_2_eq were due to travel to the injection clinic and to follow-up visits, respectively. Anti-VEGF treatment for common diseases such as age-related macular degeneration and diabetic macular edema was associated with a lower CO_2_eq compared with treatment of patients suffering from less prevalent conditions such as uveitis (10.8 kg vs. 15.6 kg). The majority of patients (49%) travelled by public transport (median distance: 22.4 km); patients travelling by car (33%) usually covered longer distances (median distance: 61.5 km).

**Conclusion:**

Travel is a meaningful contributor to the carbon footprint of IVIs. Mitigation may be achieved by various approaches, such as longer-acting therapeutic agents or bilateral injections. To implement strategies for more sustainable IVIs, ophthalmologists may be encouraged by local and national bodies to incorporate environmental criteria into their practice.

## Key messages

*****What is known***:**

- Travel-associated emissions have been considered as a key driver of intravitreal injection’s (IVI) carbon footprint; there is limited quantitative data on IVI-associated emissions.

*****What this study adds***:**

- The average travel-associated CO_2_ equivalent (CO_2_eq) for a single IVI in an urban referral centre was 11.1 kg, which is approximately equal to the daily electricity consumption of one household. Scaling this up to 1.5 million injections, as performed in Germany annually, this would equate to 16,650 tonnes of CO_2_eq per year.
- Patients receiving anti-VEGF treatment for common diseases such as age-related macular degeneration and diabetic macular edema had a lower travel-associated CO_2_eq compared to patients with less prevalent conditions such as uveitis.

## Introduction

Intravitreal injections (IVIs) are among the most frequently performed ophthalmic procedures and have revolutionised visual outcomes and quality of life particularly in patients with neovascular age-related macular degeneration (nAMD), diabetic macular edema (DME), and retinal vein occlusions (RVO). The number of IVIs has increased significantly over the last decades; considering an aging population and novel therapeutic approaches, a further growth is forecasted [1–3].

IVIs have been associated with a substantial carbon footprint; however, only few studies have assessed IVI-related emissions. Studies from New Zealand and Ireland estimated that 14.1 kg and 13.7 kg carbon dioxide equivalent (CO_2_eq), respectively, are associated with a single injection [4, 5]. Scaling this up to a country such as Germany with about 1.5 million injections annually, this would equate to 20,550–21,150 tonnes of CO_2_eq per year [6]. Travel-associated emissions have been identified as key driver of IVI-related carbon footprint, accounting for 40% and 77%, respectively, of total emissions [4, 5]. Further contributors include building energy, manufacturing and procurement of the medication, injection associated material and disposal of waste [3]. A multinational study estimated the waste of standard disposable injection sets to be 165 g, corresponding to an associated carbon footprint of 301.7 g CO_2_eq, which would add up to 453 tonnes CO_2_eq for 1.5 million injections [2].

Current data on travel-associated emissions are from countries with centralised healthcare systems with comparatively few treatment centres compared to countries such as Germany. Likewise, there is currently only little data on IVI-associated emissions from urban areas. However, the carbon footprint may highly depend on local healthcare settings and treatment guidelines. This study therefore investigated travel-associated emissions in an urban setting and in the context of a decentralised healthcare system. 

## Materials and methods

This cross-sectional study was conducted at the Department of Ophthalmology, University Medical Center Hamburg-Eppendorf (UKE), Germany. The study adhered to the declaration of Helsinki. Institutional review board approval and patients’ informed consent were obtained.

All patients received their retinal assessment and injections at the Department of Ophthalmology at the University Medical Center Hamburg-Eppendorf. Hamburg is Germany’s second-largest city and a medical reference centre in Northern Germany. Within Hamburg, the UKE is centrally located and easily accessible via public transport and well-connected to the city’s infrastructure.

Patients presenting in the injection clinic were assessed regarding the location from which they travelled to the clinic, which mode of transport they used, and whether they attended the clinic by themselves or if they were accompanied. Furthermore, patients were asked if they will attend a postoperative follow-up visit after the injection, which is recommended in Germany, and where that follow-up visit takes place. The distance travelled was assessed based on the mode of transport used. Each patient was included only once.

The distance travelled (home to hospital, home to local ophthalmologist for follow-up visit, and vice versa) was calculated using Falk route planner (Mairdumont, Ostfildern, Germany), which displays distances for every mode of transport [7]. In case patients had a driver, the distance travelled by the driver was included. Personal information regarding gender, age, treated condition and medication was collected based on electronic healthcare records.

The CO_2_eq was calculated using the full set of 2024 conversion factors as published by United Kingdom (UK)’s Department for Energy Security and Net Zero [8]. The conversion factor for an average car was calculated by taking the distribution of personal vehicles’ different fuel types in Germany in 2024 into account [9], resulting in a conversion factor of 0.16. The conversion factors listed for the “average local bus” (0.11) and for a “regular taxi” (0.21) [8] were used for calculating the carbon footprint of patient travel by public transport and by taxi/ambulance transport, respectively.

Travel-associated CO_2_eq was compared between patients receiving different intravitreal medications [(I) anti–vascular endothelial growth factor (VEGF), and (II) dexamethasone intravitreal implants, triamcinolone, and ganciclovir IVI]. Furthermore, the travel-associated CO_2_eq was compared between patients with different diagnoses [nAMD, RVO, DME, uveitis, and other conditions].

## Results

In total, 340 patients (155 female; median age 73 years; range 16–99 years) were included in this study. Treatment indication for IVIs included nAMD (*n* = 151); RVO (*n* = 77); DME (*n* = 44); secondary or idiopathic CNV (*n* = 34); cancer-associated retinal disease (*n* = 12); postoperative macular edema (*n* = 11); uveitis (*n* = 9); and other conditions (*n* = 2).

On the date of assessment, 328 patients received an IVI after evaluation of clinical symptoms, ophthalmic examination and retinal imaging. This included bevacizumab (*n* = 163), aflibercept (*n* = 89), ranibizumab (*n* = 34), faricimab (*n* = 21), dexamethasone intravitreal implant (*n* = 14), ganciclovir (*n* = 5), and triamcinolone (*n* = 1). One patient with nAMD received bevacizumab in one and aflibercept in the fellow eye. 12 patients without signs of active disease did not receive an IVI.

### Patient travel

**Fig. 1:**
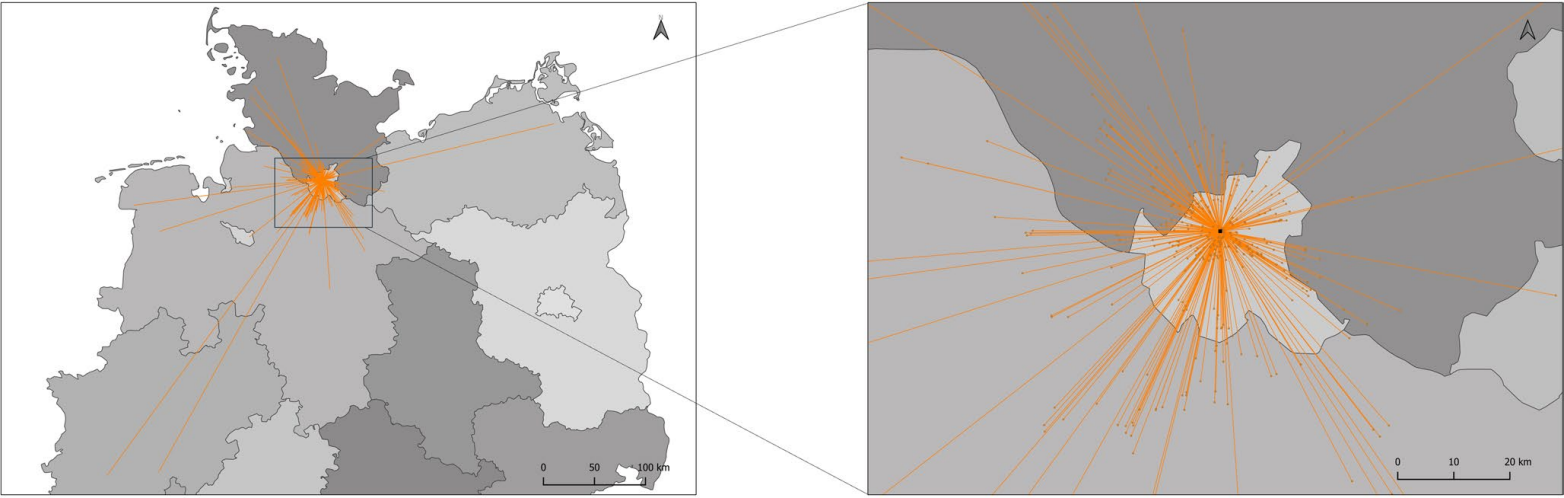
Travel for intravitreal injections at the University Medical Center Hamburg- Eppendorf, Germany. Straight-line distance between patients’ home and the injection clinic is shown over a map of Northern Germany

Patients attended the clinic by public transportation (*n* = 156), car (*n* = 104), taxi/ambulance (*n* = 49), on foot (*n* = 5), or by bicycle (*n* = 3) (Table 1). Different modes of transport were used by 23 patients; most of them took public transportation one way and returned by car (*n* = 15) or taxi/ambulance transport (*n* = 4). Four patients travelled by car for one leg of the journey and used taxi or ambulance transport for the return trip (Table 1). Linear distances between patients’ home and the injection clinic are shown in Fig. 1.

**Table 1:** Median and range of total distance to and from the injection site as well as the CO_2_ equivalent per patient

Mode of transport	Total distance		Kg CO_2_ equivalent per patient
	Median (km)	Range (km)	
Car (*n* = 104)	61.5	10.1–892	15.7
Public transport (*n* = 156)	22.4	1.4–1008	5.2
Bike (*n* = 3)	2.4	1.5–3.3	0
Walking (*n* = 5)	3.1	1.4–4.2	0
Taxi/Ambulance transport (*n* = 49)	29.6	5.3–136	9.1
Public transport and Car (*n* = 15)	31.9	8.6–276	9.4
Public transport and Taxi/Ambulance transport (*n* = 4)	42.4	11.9–90.5	7.4
Car and Taxi/Ambulance transport (*n* = 4)	35.7	31.3–70.1	7.6

Of the 104 patients travelling by car only, 90 had a driver. More than half (*n* = 56) shared the same household as the patient; the majority of drivers (*n* = 71) waited at the hospital during the injection appointment. The median distance for the return journey, including distances travelled by the driver only, was 61.5 km (range: 10.1–892 km) (Table 1). 9 patients travelled more than 200 km for the return trip; 8 of those were residents in Northern Germany, the patient travelling the longest distance (892 km) used to live in Hamburg and did not want to change the IVI clinic after his relocation.

Patients taking the taxi or ambulance transport travelled a median distance of 29.6 km (range: 5.3–136 km). The median distance for patients travelling by public transport was 22.4 km (range: 1.4–1008 km). A few of the latter (*n* = 6) travelled more than 200 km; 5 were residents in Northern Germany while the patient travelling 1008 km underwent ophthalmic surgery in Hamburg during a business trip and continued his therapy in our centre afterwards. When patients walked or cycled, distances to the clinic were usually comparably short (median walking: 3.1 km, range: 1.4–4.2 km; median cycling: 2.4 km, range: 1.5–3.3 km) (Table 1).

Of the 23 patients using different modes of transportation, median combined travelled distance was between 31.9 km (range: 8.6–276 km; car and public transport) and 42.4 km (range: 11.9–90.5 km; public transport and taxi/ambulance transport) (Table 1).

Patients receiving anti-VEGF therapy travelled shorter distances than those receiving a different treatment including dexamethasone implants, triamcinolone, or ganciclovir (median: 30.6 km vs. 43.5 km). Patients with common indications for intravitreal injections including nAMD, DME or RVO travelled shorter distances than those with rather rare diseases (nAMD: median 26.1 km; RVO: median 33.9 km; DME: median 27.8 km; uveitis: median 52.9 km; other conditions: median 42.3 km).

### Travel to follow-up visit

Of the 328 patients receiving intravitreal injections, 82% (*n* = 269) underwent a follow-up visit within the first 4 days after the intravitreal injection which was usually conducted by the local ophthalmologist. For the travel to the follow-up visit, the majority took the car (*n* = 102) or used public transport (*n* = 82); however, a large proportion of patients also walked (*n* = 56) or cycled (*n* = 18) (Table 2). Of the patients travelling by car, 50 had a driver, hereof 33 lived in the same household.

**Table 2:** Median and range of total distance to and from follow-up visit summed up as well as the CO_2_ equivalent per patient

Mode of transport	Total distance		Kg CO_2_ equivalent per patient
	Median (km)	Range (km)	
Car (*n* = 102)	16.3	1–189.6	4.8
Public transport (*n* = 82)	7.1	1.4–69.3	1.4
Bike (*n* = 18)	5	1.6–32.9	0
Walking (*n* = 56)	1.6	0.04–10.6	0
Taxi/Ambulance transport (*n* = 11)	18.6	2.6–71.6	5.6

The longest median distance to and from the follow-up visit was covered by patients taking the taxi/ambulance transport (*n* = 11) (median: 18.6 km, range: 2.6–71.6 km), followed by those travelling by car (median: 16.3 km, range: 1–189.6 km). Patients using public transport covered a median distance of 7.1 km (range: 1.4–69.3 km), while those walking travelled a median of 1.6 km (range: 0.04–10.6 km). Patients riding bicycles travelled a median of 5 km (range: 1.6–32.9 km) (Table 2).

### CO_2_ equivalent

Based on the data above, the travel-associated CO_2_eq for a single IVI was 11.1 kg CO_2_eq on average. Of this, 9.1 kg CO_2_eq and 2 kg CO_2_eq were due to travel to the injection clinic and the follow-up visit, respectively. In case of anti-VEGF treatment, travel-associated CO_2_eq was 10.8 kg while for patients receiving a dexamethasone intravitreal implant, intravitreal triamcinolone or ganciclovir it was 15.6 kg.

The most common diagnoses nAMD, RVO and DME were associated with a lower travel-associated CO_2_eq (9.9 kg, 10.3 kg, and 12.4 kg, respectively) while uveitis was associated with a higher travel-associated CO_2_eq of 14.5 kg; the remaining diagnoses were associated with a travel-associated CO_2_eq of 13.5 kg.

Depending on the mode of transport, the CO_2_eq for travel to the injection site ranged between 0 (patients biking or walking) and 15.7 kg (patients taking the car). The use of different modes of transportation was associated with a carbon footprint between 7.4 and 9.4 kg CO_2_eq (Table 1). In general, the CO_2_eq for travel to the follow-up visit was lower, ranging between 0 and 5.6 kg (Table 2).

## Discussion

The environmental impact of healthcare practices, accounting for about 5% of global emissions, has become increasingly important. Understanding and mitigating emissions of leading ophthalmic procedures appears vital for developing more sustainable practices and may underscore the pioneering role of ophthalmology in reducing healthcare-associated emissions [3, 10, 11].

Travel-related emissions have been identified as a major contributor to the carbon footprint of IVIs [3–5]. In a previous study from an urban tertiary referral centre in Dublin, Ireland, travel-related emissions were estimated to be 10.5 kg CO₂eq per IVI, which is similar to our data with 11.1 kg CO₂eq per IVI [5]. This is approximately equal to the daily electricity consumption of one household [12]. Notably, 2 kg CO₂eq in our study were associated with post-injection follow-up examinations which are not common in most countries worldwide.

In Dublin, most patients (64%) travelled by car, followed by public transport (32%). Patients using public transport covered longer distances compared to those travelling by car (average one-way distance: 51 km [bus], 42 km [train], 33 km [car]) [5]. In contrast, in our cohort, the majority of patients used public transport (49%). 33% travelled by car who usually covered longer distances than those using public transport (mean one-way distance 49 km vs. 24 km). Reasons may include that patients from rural areas are more likely to travel by car due to limited public transportation. Overall, the distance travelled by our patients appeared relatively high which could be attributed to the broad catchment area of our department. This might explain why the carbon footprint measured in our study is relatively high, even though a majority travelled by public transport.

A study from New Zealand with multiple injection-only clinics (tertiary referral centre and community injection clinics) revealed a lower travel-related carbon footprint of 5.6 kg CO₂eq per IVI, accounting for 40% of IVI-associated total emissions [4]. This difference is likely due to a shorter average travel distance (23 km), compared to our and the Irish study. Similar to the Irish cohort, most patients travelled by private vehicle, with minimal use of public or active transport (0–15%) [4, 5]. Neither of the previous studies reported on the injected medication. We speculate that mainly anti-VEGF agents were administered, while our analysis also covered further intravitreal medications, which may have influenced the overall carbon footprint. Moreover, we included distances covered by drivers which the other studies may not have done. Furthermore, the Irish and New Zealand studies reported average travel distances only; we do think that the median is more suitable, as it is less sensitive to outliers [13].

Future studies conducted in other regions including rural areas, smaller cities, and regions with varying healthcare provision could provide valuable insights into how local infrastructure affects travel-related emissions and the impact of regional healthcare accessibility on the carbon footprint of patient travel. Furthermore, our findings indicate that patients receiving treatment for rather rare conditions such as (non-)infectious uveitis travelled longer distances compared to those with frequent diagnoses like nAMD, RVO or DME and/or receiving anti-VEGF therapy. This difference may be attributed to the expertise in our reference centre, whereas the medical knowledge for common diseases is likely more widespread within the ophthalmic community. So far, data on the carbon footprint of different medications (such as anti-VEGF therapies) are not available which may be of value to incorporate into further evaluations. 

There are several possibilities to reduce the travel-associated carbon footprint of IVIs. One would be to perform more bilateral same-day IVIs, which have been shown to be safe, were preferred by patients, and had a good acceptance [14–17]. In our study, about 17% of patients required bilateral treatment, which is lower than previously reported [18]. In a UK study, bilateral injections were performed in 19.2% of visits in patients with DME and 3.0–10.4% of visits in patients with nAMD [1]. Scaling our calculation up to about 1.5 million injections which are performed in Germany annually [6], about 220,000 patients require bilateral treatment in Germany. If all of these injections were performed bilaterally on the same visit instead of unilaterally in separate visits, the annual carbon footprint could be reduced by 2,415 tonnes CO_2_eq. Even though bilateral disease is often associated with a higher need for follow-ups and bilateral same-day IVI is probably not an option for all patients, especially not for those with asymmetric treatment intervals between eyes, this option shall also be considered both from an environmental and patient perspective. Another approach is the use of longer-acting agents such as high-dose aflibercept [19, 20] or faricimab [21, 22], to reduce injection frequency. When possible, healthcare providers could prioritise treatments requiring fewer injections, not only to reduce patients’ burden but also to decrease emissions.

The majority of the investigated patients underwent follow-up examinations after IVIs. Post-injection controls are recommended in Germany 1 to 4 days after injections [23]. In other countries, no follow-up visits are recommended in general; patients are advised to immediately present if there are signs of complication or infection [24]. Even though waiving such follow-up visits may potentially reduce travel-associated emissions, it appears crucial that local healthcare considerations and patient pathways have been considered before.

A broader approach would be to consider the infrastructure of IVI clinics and public transportation, as improvements in these areas could reduce travel-associated emissions. For example, if all patients travelled by electric vehicles, the travel-associated carbon footprint per patient could be reduced by more than 40% from 11.1 to 6.4 kg CO_2_eq; when only considering patients travelling to the injection clinic by car, the carbon footprint would decrease from 15.7 to 4.6 kg CO_2_eq per patient.

Strategies for mitigating the environmental impact of IVIs should not be restricted to travel-associated emissions only. Building energy, (cold chain) logistics, manufacturing, procurement of medications and procedure related material, as well as the material used and waste disposal are components that might be modified to lower IVI’s carbon footprint [3]. For example, injection-associated waste may be significantly reduced by using a material-sparing approach without putting patients at risk [2, 25]. In general, comprehensive life cycle assessments (LCA) of IVIs appear vital which shall include information from pharmaceutical companies. LCAs including production processes shall also be performed for upcoming therapeutic approaches such as port delivery systems, sustained-release implants or gene therapies which may reduce the frequency of administration and potentially the travel-associated carbon footprint.

Limitations of this study include the single-centre approach and the focus on an urban tertiary referral centre. Additional studies including injection clinics in non-urban areas and community injection clinics are currently planned.

In conclusion, this study confirms that travel accounts for a meaningful carbon footprint of IVIs. To implement strategies for more sustainable IVIs, ophthalmologists may be encouraged by local and national bodies to incorporate environmental criteria. Healthcare organisations may support and adopt sustainability policies to reduce emissions of ophthalmic procedures.
